# The Ifng antisense RNA 1 (*IFNG-AS1*) and growth arrest-specific transcript 5 (*GAS5*) are novel diagnostic and prognostic markers involved in childhood ITP

**DOI:** 10.3389/fmolb.2022.1007347

**Published:** 2022-10-12

**Authors:** Marwa A. Ali, Sherin Khamis Hussein, Abeer A. Khalifa, Amani M. El Amin Ali, Marwa S. Farhan, Amal A. Ibrahim Amin, Esam Ali Mohamed

**Affiliations:** ^1^ Department of Medical Biochemistry and Molecular Biology, Faculty of Medicine, Fayoum University, Fayoum, Egypt; ^2^ Department of Paediatrics, Faculty of Medicine, Fayoum University, Fayoum, Egypt; ^3^ Department of Physiology, Faculty of Medicine, Zagazig University, Zagazig, Egypt; ^4^ Department of Medical Physiology, Faculty of Medicine, Fayoum University, Fayoum, Egypt; ^5^ Department of Clinical and Chemical Pathology, Faculty of Medicine, Cairo University, Cairo, Egypt; ^6^ Department of Medical Microbiology and Immunology, Faculty of Medicine, Fayoum University, Fayoum, Egypt

**Keywords:** *IFNG-AS1*, *GAS5*, childhood ITP, qRT-PCR, diagnosis, prognosis, biomarkers

## Abstract

**Background/aim:** *IFNG-AS1* is a long noncoding RNA that works as an enhancer for the Interferon-gamma (IFN-γ) transcript. *GAS5* (growth arrest-specific 5) is a lncRNA that is associated with glucocorticoid resistance. Aberrant expressions of *IFNG-AS1* and *GAS5* are directly linked to numerous autoimmune disorders but their levels in childhood ITP are still obscure. This study aims to elucidate expressions of target lncRNAs in childhood ITP and their association with pathophysiology and clinical features of the disease as well as their association with types and treatment responses.

**Method:** The fold changes of target lncRNAs in blood samples from children with ITP and healthy controls were analyzed using quantitative real-time PCR (qRT-PCR).

**Results:** There were overexpressed lncRNAs *IFNG-AS1* and *GAS5* in serum of childhood ITP patients [(median (IQR) = 3.08 (0.2–22.39) and 4.19 (0.9–16.91) respectively, Also, significant higher *IFNG-AS1* and *GAS5* (*p* < 0.05) were present in persistent ITP (3–12 months) [ median (IQR) = 4.58 (0.31–22.39) and 3.77 (0.87–12.36) respectively] or chronic ITP (>12 months) [ median (IQR) = 5.6 (0.25–12.59) and 5.61 (1.15–16.91) respectively] when compared to newly diagnosed <3 months patients [*IFNG-AS1* median (IQR) = 1.21 (0.2–8.95), and *GAS5* median (IQR) = 1.07 (0.09–3.55)]. Also, significant higher lncRNAs *IFNG-AS1* and *GAS5* were present in patients with partial response to treatment [*IFNG-AS1* median (IQR) = 4.15 (0.94–19.25), and *GAS5* (median (IQR) = 4.25 (0.81–16.91)] or non-response [*IFNG-AS1* median (IQR) = 4.19 (1.25–22.39) and *GAS5* median (IQR) = 5.11 (2.34–15.27)] when compared to patients who completely responded to treatment (*IFNG-AS1* median (IQR) = 2.09 (0.2–14.58) and *GAS5* (median (IQR) = 2.51 (0.09–10.33). In addition, following therapy, the expressions of *IFNG-AS1* and *GAS5* are significantly negatively correlated with platelet count.

**Conclusion:** Findings suggest that lncRNAs *IFNG-AS1* and *GAS5* are novel diagnostic and prognostic genetic markers for childhood ITP that can aid in a precise prediction of the disease’s progress at the time of diagnosis and could be a useful tool for treatment planning.

## Introduction

A vast number of non-coding transcripts were discovered during human genome sequencing, they were formerly believed to be non-functional, however recently, scientists discovered that they have regulatory functions on gene expression involving transcriptional, posttranscriptional, and translational levels. Long noncoding RNAs (lncRNAs) are one type of them which defines as RNA transcripts with more than 200 nucleotides that are not translated into protein (Palazzo and Lee, 2015). As more evidence accumulates, lncRNAs appear to have an essential function in immune control, suggesting that aberrant lncRNAs expressions may have played a part in the triggering of autoimmunity comes in a variety of forms (Gao et al., 2018).

*IFNG-AS1* (Ifng antisense RNA 1), also known as *Tmevpg1* (Theiler’s murine encephalomyelitis virus persistence candidate gene 1), or *NeST*, (nettoie *Salmonella* pas Theiler’s), is affiliated as lncRNA transcript which gene is found on the DNA strand opposite the interferon-gamma (IFN- γ) coding gene so hence its name. The coexpression of lncRNA, *IFNG-AS1* with T-bet is related to the active transcription of IFN- γ in Th1 cells. Th1 cells are an inflammatory fraction of CD4^+^ T cells that primarily generate interferon-gamma (IFN-γ), an inflammatory cytokine that protects against intracellular pathogens, and is related to delayed-type hypersensitivity, and autoimmune diseases including ITP (Li et al., 2016), (Collier et al., 2012a).

*GAS5* (growth arrest-specific 5) is a lncRNA that was first discovered as a cancer tumor suppressor gene. *GAS5* expression has been found to be abnormal in numerous malignancies, and this gene has been linked to cell cycle inhibition and apoptosis (Ji et al., 2019). Recently, scientists discovered that *GAS5* dysregulation has been implicated in the development of autoimmunity states; The most effective medications for treating autoimmune illnesses are glucocorticoids (GC). *GAS5* interacts with the DNA binding domain of glucocorticoid receptors (GRs), and preventing GRs from connecting with DNA, preventing glucocorticoid activity which is a powerful immunosuppressive and hence contributes to the development of autoimmune disorders (Moharamoghli et al., 2019).

Immune thrombocytopenic purpura (ITP) is a blood disorder characterized by a decreased platelet count below 100×10^9^/L with normal hemoglobin and leucocytes, when there are no additional causes or conditions that could be linked to thrombocytopenia, the condition is called idiopathic or primary ITP. The fundamental immunologic abnormality in ITP is thought to be humoral and cell-mediated antiplatelet antibodies thus leading to enhanced platelet destruction and often inappropriate platelet manufacture in the bone marrow. These phenotypic changes reflected a change in gene regulation (Makis et al., 2017), (Ayoub et al., 2020).

Childhood Immune Thrombocytopenia (ITP) is one of the most frequent autoimmune hemorrhagic diseases affecting 5 to 10 children out of every 100,000. Twenty to thirty percent of cases were shown to be prone to chronicity with increased susceptibility to bleeding, infection, and blood malignancy than healthy children (Beshir et al., 2021) (Li et al., 2020). Previous studies demonstrated that when compared to children who received therapeutic regimens, the no therapy group had a significantly higher recurrence and more serious complications (Makis et al., 2017) (Kühne et al., 2011). The lncRNAs involved in immune mechanisms that play role in the initiation of the disease are the targets of therapy when indicated, Aberrant *IFNG-AS1* and *GAS5* expressions are linked to numerous autoimmune disorders suggesting the possible role of these genes in the pathogenesis of autoimmunity state (Li et al., 2018).

There are two types of ITP; Adult ITP and childhood ITP (Kühne et al., 2011), although, two separate studies have reported that levels of *IFNG-AS1and GAS5* in peripheral blood of adult ITP patients (Li et al., 2016), (Li et al., 2020), the expressions of *IFNG-AS1* and *GAS5* in childhood ITP are still unknown. Thus, we designated this study to investigate the expressions of lncRNAs *IFNG-AS1* and *GAS5* in childhood ITP and their relationship to clinical characteristics of the disease, as well as types and treatments.

## Materials and methods

### Materials

Materials used to execute this work include; one- MiRNeasy Serum/Plasma extraction kit (Qiagen, Valencia, CA, USA) was used for total RNA extraction from serum. Two- The RT2 First Strand Kit (Qiagen, Valencia, CA, USA) was used for reverse transcription to produce cDNA. Three- The RT2 SYBR Green PCR kit (Qiagen, Maryland, USA) was used to perform qRT-PCR along with specific primers supplied by Qiagen, (Valencia, CA, USA); for *IFNG-AS1* (Catalog no; 330701LPH20079A, Accession no, ENST00000536914.0) and *GAS5* (Catalog no; 330701LPH11340A, Accession no, NR_002578.2) and we used GAPDH primer (Catalog no: 330701 LPH31725A, Accession no: ENST00000496049.0) to standardize the expression pattern and quantify the target long non-coding RNAs.

### Patients and controls

This case-control study was comprised of eighty-eight children (1–13 years old) who suffered from primary ITP and eighty-eight healthy age and sex-matched children with no history of any disease. Cases were gathered from the Pediatric Departments’ inpatient and outpatient clinics at Fayoum University Hospital in Egypt over a period of 6 months between Jan 2022 and June 2022.

### Diagnostic criteria

A detailed history was taken including age, sex, complaint at presentation, disease duration, presence of bleeding manifestations, history of splenectomy, presence of preceding febrile illness, recent vaccination, *helicobacter pylori* infection history, HCV and/or HBV viral infection. Causes of secondary ITP such as autoimmune rheumatological diseases and the previous treatment regimen. A systematic clinical examination is performed, with special attention paid to clinical symptoms of bleeding such as bruises or purplish spots on the skin, mucous membranes, or gums, and any concomitant splenomegaly. Laboratory tests including complete blood count (CBC) and bone marrow examination to exclude other diseases resulting in thrombocytopenia were done. The definite diagnosis was established as primary idiopathic thrombocytopenia depending on the presence of solitary low platelet count (<100×10^9^/L) in a patient with multiple bruises and/or petechiae.

### Types of ITP and response to treatment

The ITP patients were categorized regarding the duration of illness, 42 (47.72%) were newly diagnosed <3 months, 27 (30.86%) with persistent ITP 3–12 months, and 19 (21.60%) had chronic >12 months ITP. Treatment options regimen includes corticosteroids, IVIG, immunosuppressive drugs, and Eltrombopag (detailed treatment regimens are presented in Table 2). Response to treatment; 38 (43.18%) of patients showed complete response to treatment (CR) which is defined as platelet count ≥100×10^9^/L without clinically significant bleeding, 43 (48.87%) showed partial response to treatment (PR); which is a platelet count between 30 and 100×10^9^/L or twice the baseline platelet count without significant bleeding, and 7 (7.95%) with no response (NR) in patients with the present spleen is platelet count <30×10^9^/L in two different measurements or the increase in platelet is less than twice baseline count (Makis et al., 2017)., (Rodeghiero et al., 2009)

### Inclusion and exclusion criteria

Children aged 1–13 years, of both sexes, with primary ITP were included in the study while patients with secondary causes for purpura, splenectomy, with concurrent viral, or autoimmune diseases were excluded from the study or with a history of receiving medications cause thrombocytopenia.

### Ethical consideration

Before the sample was taken, the study protocols were thoroughly explained to the parents, and they were asked to sign an informed consent form. The Ethics Committee at the Faculty of Medicine in Fayoum University reviewed and approved this work protocol no (R210-89). This work was done per the Declaration of Helsinki’s ethical requirements.

### Handling of sample

With the help of skilled medical staff, we withdrew 5 mL of venous blood from each participant into a plain tube for serum preparation and RNA extraction. For serum separation, samples were left on a straight surface for nearly 15 min, supernatants were transferred to clean tubes and centrifuged at 4,000 xg for 10 min to isolate serum which was kept at -80°C until use.

### RNA extraction and assessment of its purification

Total RNA was extracted from the serum according to the manufacturer’s instructions using the miRNeasy Serum/Plasma extraction kit (Qiagen, Valencia, CA, USA). The first step is to clean the area of work, pipettes, and centrifuge with prepared ethanol 70%, second step is aimed at lysis of cells and release of nucleic acid so after wearing clean gloves we added 1,000 μl QIAzol lysis reagent to 200 μL sample and incubated the mix after shaking vigorously at room temperature for 5 min. Thirdly, to enhance phase separation so that RNA is purified from DNA and protein debris, we added 200 μl chloroform, vortexed, and put the samples in the freezer for 5 min to avoid RNA destruction then the samples were centrifuged at 12.000 xg for 15 min. Then, we collected the upper aqueous parts in new tubes and added 1.5% of its volume to 100% ethanol to promote RNA precipitation. Afterward, we used the mini spin column in a 2 ml collection tube accompanied RNeasy kits to promote solid phase separation of RNA by adding the samples into two subsequent sessions (750 μl) each with centrifugation at 8,000 *g* for 15 s, discarded the flow-through and transferred the spin column containing binding RNA to clean 2 ml collection tubes. We used the washing buffers RWT and RPF in the kit; each spin column received 700 μl of RWT buffer, then centrifuged at 8,000 xg for (Fouad et al., 2022a) s, the flow-through was discarded, and the column was reused for the following phase, we next pipetted a 500 μl buffer RPE to the spin column and centrifuged it at 8,000 xg for 15s, discarded the flow-through water, and repeated the previous process. We moved the spin column to a fresh collecting tube and centrifuged it at full speed for 2 min to dehydrate RNA samples. Finally, for elusion, the spin column was transferred to a clean Eppendorf tube and 50 ul Rnase-free water was pipetted directly onto the column before centrifugation at 8,000 xg for 1 min. We next used a NanoDrop 1,000 spectrophotometer (Thermo Scientific, Waltham, MA, USA) to measure the RNA concentration and purification at 260/280 nm A ratio of 2.0 was considered “pure.”

### Reverse transcription (RT) of RNA into complementary DNAs (cDNAs)

The RT2 First Strand Kit (Qiagen, Valencia, CA, USA) was used for reverse transcription to produce cDNA, and the manufacturer’s procedure was followed. RT reaction was done in a final volume of 20 μl (10 μl reverse-transcription mix was added to each tube containing genomic DNA elimination mix), Conventional PCR was used to incubate the samples for 60 min at 37°C. followed by incubation for 5 min at 95°C to inactivate reverse transcriptase.

### Quantitative real-time PCR (qPCR) for detection of long non-coding RNAs

It has previously been shown that lncRNAs were expressed in serum (Mohammed et al., 2022)– (Visconti et al., 2020). The RT2 SYBR Green PCR kit (Qiagen, Maryland, USA) was used to evaluate the expression of the lncRNAs *IFNG-AS1* and *GAS5* in serum using specific primers supplied by Qiagen, (Valencia, CA, USA); for *IFNG-AS1* (Catalog no; 330701LPH20079A, Accession no, ENST00000536914.0) and *GAS5* (Catalog no; 330701LPH11340A, Accession no, NR_002578.2) and we used GAPDH primer (Catalog no: 330701 LPH31725A, Accession no: ENST00000496049.0) to standardize the expression pattern and quantify the target long non-coding RNAs (Li et al., 2016), (Peng et al., 2021). The reaction mix was prepared in a nuclease-free tube according to the manufacturer’s protocol for a 25 μl per well reaction volume. The Rotor-gene Q Real-time PCR system (Qiagen, USA) was used to perform quantitative real-time PCR under the following conditions: 95°C for 10 min, then 45 cycles of 15s at 95 and 60°C for 1 min.

### Calculation of results

Melting curve tests were carried out after the PCR cycles were completed to confirm the particular production of the expected PCR result. The cycle threshold (Ct) value is the number of qPCR cycles essential to the fluorescent signal to cross a specific threshold. By deducting the Ct values of GAPDH from those of the target long non-coding RNAs, Δ*Ct* was determined, and by subtracting the Δ*Ct* of the control samples from the Δ*Ct* of the cases samples, ΔΔ*Ct* was determined. Then we used the subsequent equation (2^−ΔΔCt^ equation) (Livak and Schmittgen, 2001) to demonstrate the fold change of target genes relative to controls which were set as 1.

### Statistical analysis

Data was presented by mean ± SD (Standard Deviation), number and percentage, median and interquartile range; (IQR). SPSS version 22 (SPSS Inc) was used for analyzing data. The mean, SD, median, and range were calculated for the quantitative data. One-way ANOVA test was used for normal distribution data analysis in more than two groups. When variables were not normally distributed, the Mann–Whitney-U test (2 groups) or Kruskal Wallis test (more than two groups) was used in comparing groups. Otherwise, the independent-T test was used. The significance of the qualitative data was detected by chi-square (χ2). Pearson correlation was done to explore the association between *IFNG-AS1* and *GAS5* and the clinical parameters, treatment, and response to treatment. The sensitivity and specificity of *IFNG-AS1* and *GAS5* each alone or combined regarding the discrimination between ITP cases and healthy control subjects the receiver operating characteristic (ROC) curve analysis was done. All the results of the tests were interpreted as significant by considering that *p* ≤ 0.05.

### Sample size calculation

The sample size equals 88 cases, to adapt the cost of research without decreasing the validity of results we used the formula;

Necessary sample size = $\frac{\left(Z-Score\right)2\;X\;SD\;X\left(1-SD\right)}{\left(margin\;of\;error\right)2}$

to get the appropriate sample size involved in this research, confidence levels 95% were converted into Z scores, the standard deviation was 0.5, and the margin of error was 0.05).

## Results

### Comparison between ITP children and healthy control children regarding gender, age, and some blood smear parameters

A total of 88 ITP children, ranging in age from 1 to 13 years were included in this case-control study with mean ± SD = 4.9 ± 2.15 years and 49 (55.68%) patients were females and 88 healthy children with ages ranging from 2 to 12 years with mean ± SD = 5.1 ± 2.09 years and 45 (51.13%) were females. This study was constructed to see how effective the lncRNAs *IFNG-AS1* and *GAS5* were at distinguishing between children within ITP and those within the control group and to explore the link between their levels and types of childhood ITP as well as with treatment regimens and response.

Results represented in (Table 1) showed that the studied groups were matched regarding gender (*p* = 0.254) and age (*p* = 0.172). Regards CBC picture, ITP patients had lower hemoglobin (HB) (mean ± SD = 9.9 ± 2.19 gm%), lower platelet count (PL) either before (mean ± SD = 9.33 ± 2.51× 10^3^) or after treatment (mean ± SD = 129.14 ± 48.95 × 10^3^) and higher absolute lymphocytic count (ALC) (mean ± SD = 2,509 ± 926/ml) than controls (mean ± SD = 11.01 ± 1.58 gm% for HB, 191.35 ± 30.15 × 10^3^ for PL count, and 1815 ± 875/ml for ALC, *p* < 0.05).

**TABLE 1:** Comparison between ITP children and healthy control children regarding gender, age, and some blood smear parameters.

Parameters		Control (88)	ITP (88)	*p*-value
**Gender**	**Female**	45 (51.13%)	49 (55.68)	0.254
	**Male**	43 (48.87%)	39 (44.32)	
**Age (years)**		5.1 ± 2.09	4.9 ± 2.15	0.172
**CBC**	**HB (gm%)**	11.01 ± 1.58	9.9 ± 2.19	**< 0.001**
	**WBCs/ml**	7,958 ± 1,250	8,073 ± 1,352	0.095
	**ALC/ml**	1815 ± 875	2,509 ± 926	**0.01**
	**PL** ^**b**^ **×10** ^**3**^	191.35 ± 30.15	9.33 ± 2.51	**< 0.001**
		**ITP (88) Before treatment**	**ITP (88) After treatment**	***p*-value**
	**PL** ^**b**^ **×10** ^**3**^	9.33 ± 2.51	129.14 ± 48.95	**< 0.001**
		**Control (88)**	**ITP (88)** **After treatment**	***p*-value**
	**PL** ^**a**^ **×10** ^**3**^	191.35 ± 30.15	129.14 ± 48.95	**0.03**

**ALC**: absolute lymphocyte count, **PL** ^**b**^ **:** platelet before treatment, and **PL** ^**a**^ **:** platelet after treatment.

There was no significant difference existed between the two groups regards white blood cell count (WBC) (mean ± SD = 8,073 ± 1,352/ml for ITP patients and 7,958 ± 1,250/ml for controls, *p* = 0.095).

### The details of ITP group clinical characters concerning family history, duration of disease, symptoms, treatment, and response to treatment

The patients were divided into groups based on the duration of illness; 42 (47.72%) were newly diagnosed <3 months, 27 (30.86%) with persistent ITP 3–12 months, and 19 (21.60%) had chronic >12 months ITP. Out of 88 patients; 5 (5.68%) with positive family history, 31 (35.23%) with history of bleeding, 59 (67.05%) with preceding febrile illness, 5 (5.68%) with *helicobacter* infection, and 5 (5.68%) with splenomegaly. All patients had no history of splenectomy, hepatitis B virus (HBV), or hepatitis C virus (HCV) infection. Treatment regimens and responses to treatment are shown in (Table 2).

**TABLE 2:** The details of ITP group clinical characters concerning family history, duration of disease, symptoms, treatment, and response to treatment.

Parameter		N (%)
**Family history**	**Yes**	5 (5.68)
	**No**	83 (94.32)
**Duration of disease**	**< 3 months**	42 (47.72)
	**3–12 months**	27 (30.68)
	**> 12 months**	19 (21.60)
**PFI**	**Yes**	59 (67.05)
	**No**	29 (32.95)
**Splenomegaly**	**Yes**	5 (5.68)
	**No**	83 (94.32)
**Splenectomy**	**Yes**	0.0 (0.0%)
	**No**	88 (100%)
**Bleeding**	**Yes**	31 (35.23)
	**No**	57 (64.77)
**Helicobacter**	**Positive**	5 (5.68)
	**Negative**	83 (94.32)
**HBV**	**Yes**	0.0 (0.0%)
	**No**	88 (100%)
**HCV**	**Yes**	0.0 (0.0%)
	**No**	88 (100%)
**Treatment**	**A-Steroid**	54 (61.37)
	**B- Steroid + IVIG**	14 (15.91)
	**C- Steroid +** **ImS** **+ IVIG.**	5 (5.68)
	**D- Steroid +** **ImS**	11 (12.50)
	**E- Steroid + IVIG +** **ImS** **+ Eltrombopag**	4 (4.54)
**Response to treatment**	**A- CR**	38 (43.18)
	**B- PR**	43 (48.87)
	**C- NR**	7 (7.95)

**PFI**: Preceded by febrile illness, **HBV**: Hepatitis B virus, **HCV**: Hepatitis C virus, **IVIG:** IV immune globulin, **ImS:** Immunosuppressive, **CR:** Complete response, **PR:** Partial remission, **NR:** No response.

### The relative expressions of the serum fold change of target lncRNAs (*IFNG-AS1* and *GAS5*) in ITP patients in comparison with controls

**FIGURE 1:**
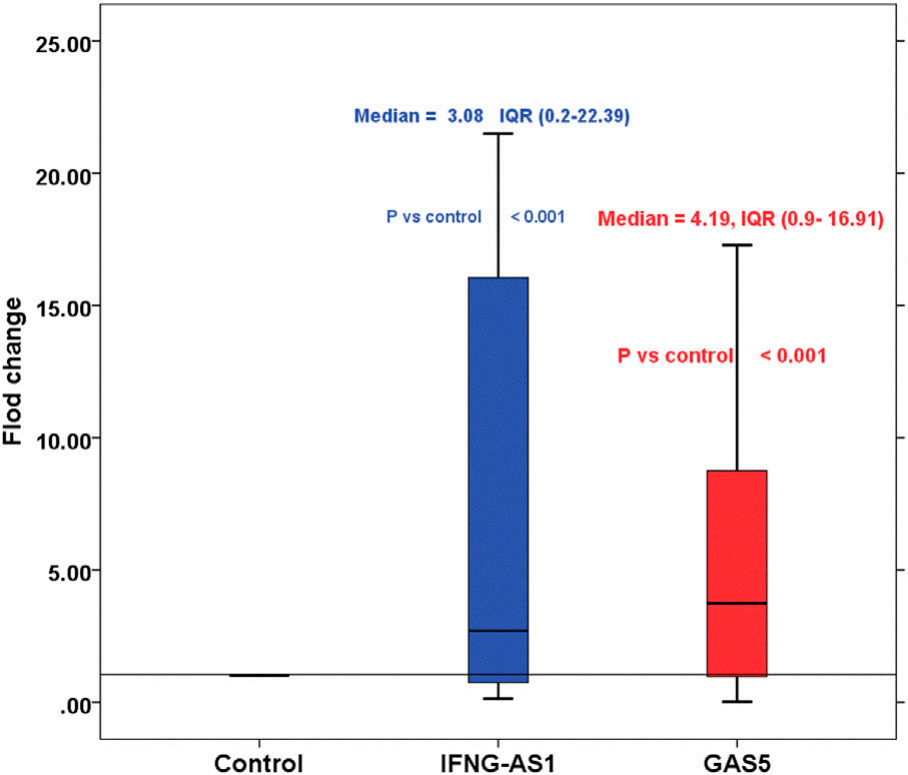
Boxplot represents the fold changes of lncRNA *IFNG-AS1* and *GAS5* in the immune thrombocytopenia (ITP) group in comparison with the healthy control group. *IFNG-AS1*: Mean ± SD = 18.37 ± 19.54, median (IQR) = 3.08 (0.2–22.39), *GAS5*: Mean ± SD = 6.17 ± 11.33, median (IQR) = 4.19 (0.9–16.91). Control was set as one according to 2^−ΔΔCt^ equation.

Children with ITP had significantly higher *IFNG-AS1* [(median (IQR) = 3.08 (0.2–22.39), mean ± SD = 18.37 ± 19.54] and *GAS5* [(median (IQR) = 4.19 (0.9–16.91), mean ± SD = 6.17 ± 11.33)] expressions in serum than that of healthy controls (*p* < 0.001 each) (Figure 1).

### The association between *IFNG-AS1* and *GAS5* expressions and the clinical signs, treatment, and response to treatment in the ITP group of patients

Regards the association between these genes and clinical characteristics, treatment and response to treatment in cases, we demonstrated that significant higher *IFNG-AS1* and *GAS5* (*p* < 0.05) were present in persistent ITP (3–12 months) [ median (IQR) = 4.58 (0.31–22.39) for *IFNG-AS1* and 3.77 (0.87–12.36) for *GAS5*] or chronic ITP (>12 months) [ median (IQR) = 5.6 (0.25–12.59) for *IFNG-AS1* and 5.61 (1.15–16.91) for *GAS5*] when compared to newly diagnosed <3 months patients [*IFNG-AS1* median (IQR) = 1.21 (0.2–8.95), and *GAS5* median (IQR) = 1.07 (0.09–3.55)]. Also, significant higher lncRNAs *IFNG-AS1* and *GAS5* were present in patients with partial response to treatment [*IFNG-AS1* median (IQR) = 4.15 (0.94–19.25), and *GAS5* (median (IQR) = 4.25 (0.81–16.91)] or non-response [*IFNG-AS1* median (IQR) = 4.19 (1.25–22.39) and *GAS5* median (IQR) = 5.11 (2.34–15.27)] when compared to patients with complete response to treatment (*IFNG-AS1* median (IQR) = 2.09 (0.2–14.58) and *GAS5* (median (IQR) = 2.51 (0.09–10.33). As well, significant high *IFNG-AS1* was detected in patients with positive family history (median (IQR) = 9.09 (2.54–22.39) than in those with negative family history (median (IQR) = 1.98 (0.2–8.04), *p* = 0.04), just significant *p*-value = 0.05 was detected between positive family history patients and negative family history patients regards *GAS5.*

In the cases group, there were no significant variations in target gene expressions in terms of gender, splenomegaly, a history of previous febrile illness or bleeding, *helicobacter* infection, or treatment regimens (Table 3).

**TABLE 3:** The association between *IFNG-AS1* and *GAS5* expressions and the clinical signs, treatment, and response to treatment in the ITP group of patients.

Variable		*IFNG-AS1*			*GAS5*		
		Mean ± SD	Median (IQR)	P	Mean ± SD	Median (IQR)	P
**Family history**	**Yes**	20.37 ± 16.4	9.09 (2.54–22.39)	**0.041**	8.54 ± 9.04	7.54 (0.2–16.91)	**0.050**
	**No**	11.25 ± 10.91	1.98 (0.2–8.04)		4.25 ± 7.25	2.38 (0.09–9.53)	
**Gender**	**Female**	19.27 ± 17.58	3.25 (0.13–22.39)	0.222	5.66 ± 9.47	4.8 (0.09–11.52)	0.351
	**Male**	16.49 ± 13.5	5.51 (0.09–15.08)		7.14 ± 9.03	3.54 (0.13–16.91)	
**Duration of disease**	**< 3 months**	6.55 ± 5.09	1.21 (0.2–8.95)	**0.002**	1.15 ± 3.07	1.07 (0.09–3.55)	**<0.001**
	**3–12 months**	9.12 ± 9.58	4.58 (0.31–22.39)		3.89 ± 4.21	3.77 (0.87–12.36)	
	**> 12 months**	19.54 ± 14.77	5.6 (0.25–12.59)		8.33 ± 7.84	5.61 (1.15–16.91)	
**PFI**	**Yes**	16.54 ± 13.35	3.56 (0.2–20.54)	0.763	8.18 ± 9.77	3.61 (0.09–17.92)	0.183
	**No**	17.15 ± 11.54	3.08 (0.54–22.39)		5.68 ± 10.23	5.34 (0.53–16.91)	
**Splenomegaly**	**Yes**	15.56 ± 11.05	3.25 (02–17.43)	0.219	4.28 ± 13.60	4.38 (1.47–9.92)	0.173
	**No**	19.18 ± 10.33	4.33 (0.99–22.39)		6.47 ± 14.58	3.95 (0.86–9.33)	
**Bleeding**	**Yes**	14.95 ± 15.20	3.08 (0.32–20.54)	0.571	6.98 ± 10.22	3.61 (0.09–16.91)	0.091
	**No**	22.35 ± 16.52	3.41 (0.2–22.39)		7.06 ± 9.92	5.91 (0.24–15.29)	
**Helicobacter**	**Positive**	17.25 ± 16.33	3.25 (0.41–20.51	0.209	7.18 ± 8.19	4.25 (0.09–16.91	0.174
	**Negative**	20.14 ± 62.5	4.02 (0.2–22.39		6.09 ± 7.55	3.87 (0.18–15.64	
**Treatment**	**A-Steroid**	9.25 ± 9.33	3.98 (0.15–18.09)	0.051	3.59 ± 5.01	3.02 (0.71–8.27)	0.065
	**B- Steroid + IVIG**	7.06 ± 7.97	3.08 (0.39–12.54)		3.66 ± 5.22	4.19 (0.09–15.64)	
	**C- Steroid +** **ImS** **+ IVIG.**	12.15 ± 9.43	4.15 (0.64–18.24)		4.25 ± 4.09	4.19 (0.2–11.77)	
	**D- Steroid +** **ImS**	16.22 ± 9.28	5.32 (0.97–22.39)		6.91 ± 4.45	5.01 (0.9–16.91)	
	**E- Steroid + IVIG +** **ImS** **+ Eltrombopag**	14.25 ± 25.24	3.14 (0.2–19.52)		3.17 ± 5.03	4.27 (0.89–17.03)	
**Response to treatment**	**A- CR**	6.09 ± 8.25	2.09 (0.2–14.58)	**0.002**	3.54 ± 3.55	2.51 (0.09–10.33)	**0.03**
	**B- PR**	13.24 ± 10.27	4.15 (0.94–19.25)		8.39 ± 7.71	4.25 (0.81–16.91)	
	**C- NR**	19.01 ± 7.14	4.19 (1.25–22.39)		9.34 ± 5.48	5.11 (2.34–15.27)	

**PFI**: preceded by febrile Illness, **IVIG:** IV, immune globulin, **ImS:** Immunosuppressive, **CR:** complete response, **PR:** partial remission, **NR**: no response.

### Pearson correlation analysis of the expression of *IFNG-AS1* and *GAS5* with age and blood smear parameters in cases

**FIGURE 2:**
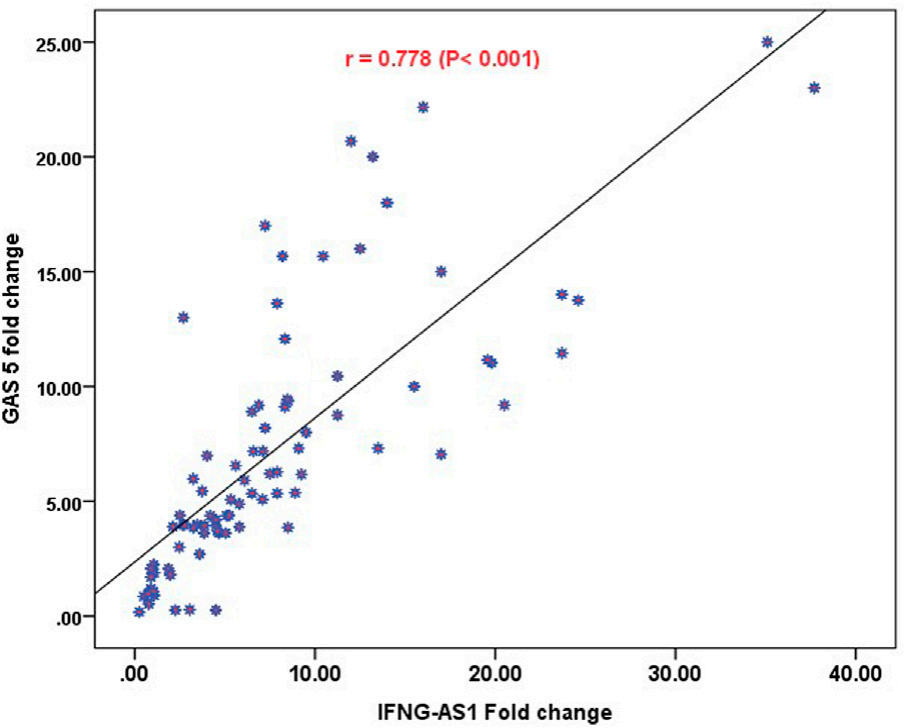
Pearson correlation analysis of the expression of *IFNG-AS1* and *GAS5*. A strong significant positive correlation was detected between *IFNG-AS1* and *GAS5* (*r* = 0.778, *p < 0.001*).

**FIGURE 3:**
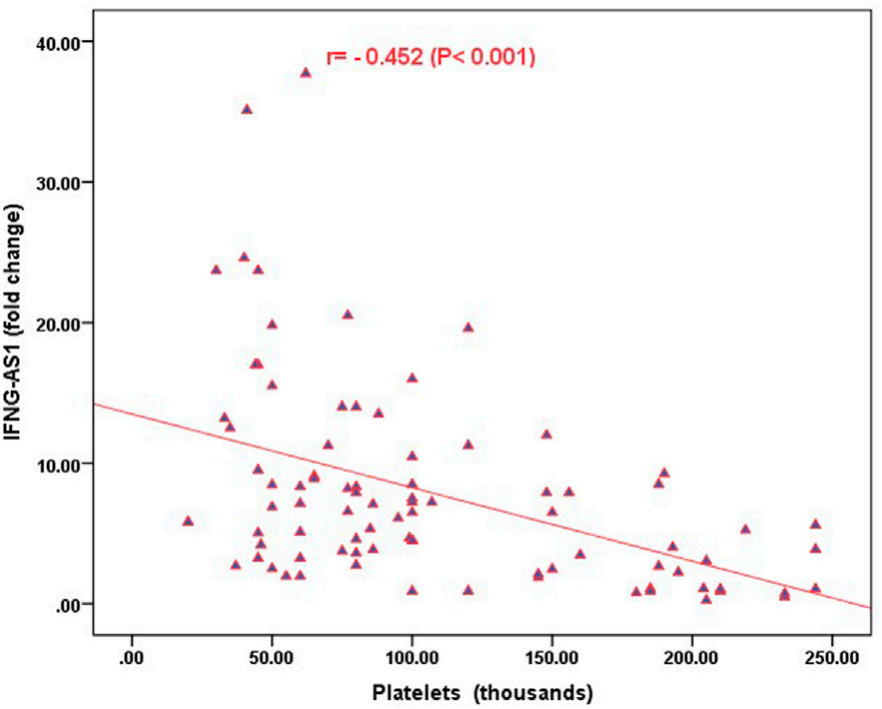
Correlation analysis between the expression of *IFNG-AS1* and platelet count after treatment. There was a negative correlation between *IFNG-AS1* fold change and platelet count after treatment (*r* = -0.452, *p <* 0.001).

**FIGURE 4:**
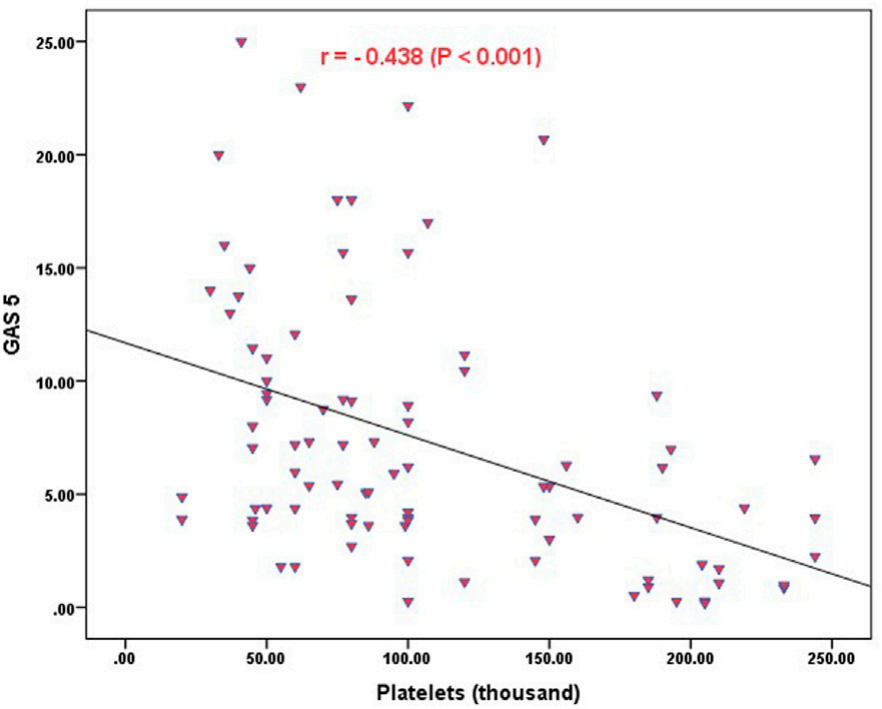
Correlation analysis between the expression of *GAS5* and platelet count after treatment. There was a negative correlation between *GAS5* fold change and platelet count after treatment (*r* = -0.438, *p <* 0.001).

Strong significant positive correlation was detected between *IFNG-AS1* and *GAS5* (*r* = 0.778, *p < 0.001*) (Figure 2), while negative correlations were detected between both genes and platelet count after treatment (*r* = -0.452, *p <* 0.001 for *IFNG-AS1* and *r* = -0.438, *p* < 0.001 for *GAS5*) (Figures 3, 4). There was no correlation between target genes and age, HB concentration, WBCs count, ALC, or PL count before treatment (Table 4).

**TABLE 4:** Pearson correlation analysis of the expression of *IFNG-AS1* and *GAS5* with age and blood smear parameters.

Parameter	*IFNG-AS1*		*GAS5*	
	*r*	*p*-value	*r*	*p*-value
***GAS5***	**0.778**	**<0.001**		
**Age**	0.1308	0.0957	0.1087	0.1541
**HB (gm%)**	0.1077	0.1333	0.0987	0.345
**WBCs/ml**	0.129	0.1008	0.118	0.153
**ALC/ml**	0.0873	0.091	0.1009	0.093
**PL** ^**b**^ **X10** ^**3**^	0.184	0.092	0.201	0.064
**PL** ^**a**^ **X10** ^**3**^	**- 0.452**	**< 0.001**	**- 0.438**	**< 0.001**

### ROC (Receiver Operating Characteristic) curve analysis to predict diagnostic values of target genes in differentiating ITP patients from controls

**FIGURE 5:**
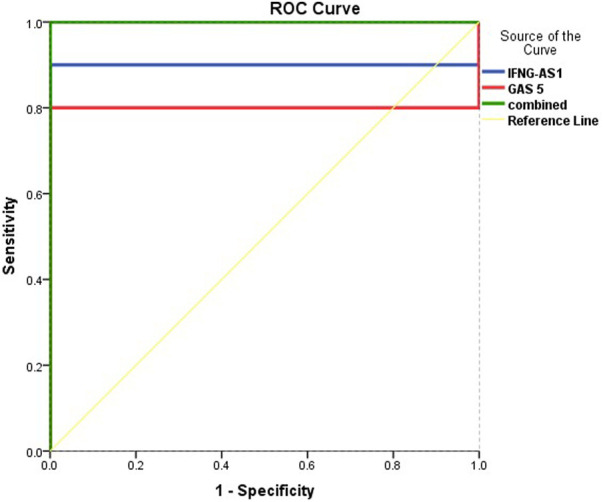
ROC curve analysis to predict diagnostic values of target genes in differentiating ITP patients from controls.

Figure 5 and Table 5 are shown the ROC curves of lncRNAs *IFNG-AS1* and *GAS5* in ITP patients.

**TABLE 5:** ROC curve analysis to predict diagnostic values of target genes in differentiating ITP patients from controls.

Variable	AUC (95% CI)	Cutoff point	*p*-value	Sensitivity (%)	Specificity (%)	Total accuracy
***IFNG-AS1***	0.900 (0.769–0.98)	1.38	**< 0.001**	66.3	97.8	82.05
***GAS5***	0.803 (0.625–0.975)	2.58	**0.001**	68.5	95.2	81.85
**Combined**	1.00	0.21	**< 0.001**	100	100	100%

ROC: Receiver Operating Characteristic, AUC: Area Under the ROC Curve, CI: Confidence Interval.

The ROC curves of lncRNAs *IFNG-AS1* and *GAS5* in ITP patients are demonstrating the diagnostic utility of these markers as predictors in distinguishing between patients with ITP and controls. LncRNA *IFNG-AS1*; AUC (95% CI) = 0.900 (0.769–0.980), *p <* 0.001, cut off point 1.38, sensitivity 66.3%, specificity 97.8%, total accuracy, 82.05%. Lnc RNA *GAS5*, AUC (95% CI) = 0.803 (0.625–0.975), *p =* 0.001, cut off point 2.58, sensitivity 68.5%, specificity 95.2%, total accuracy 81.85% and their combined expression AUC = 1.000, *p* < 0.001, sensitivity and specificity 100.0%.

**FIGURE 6:**
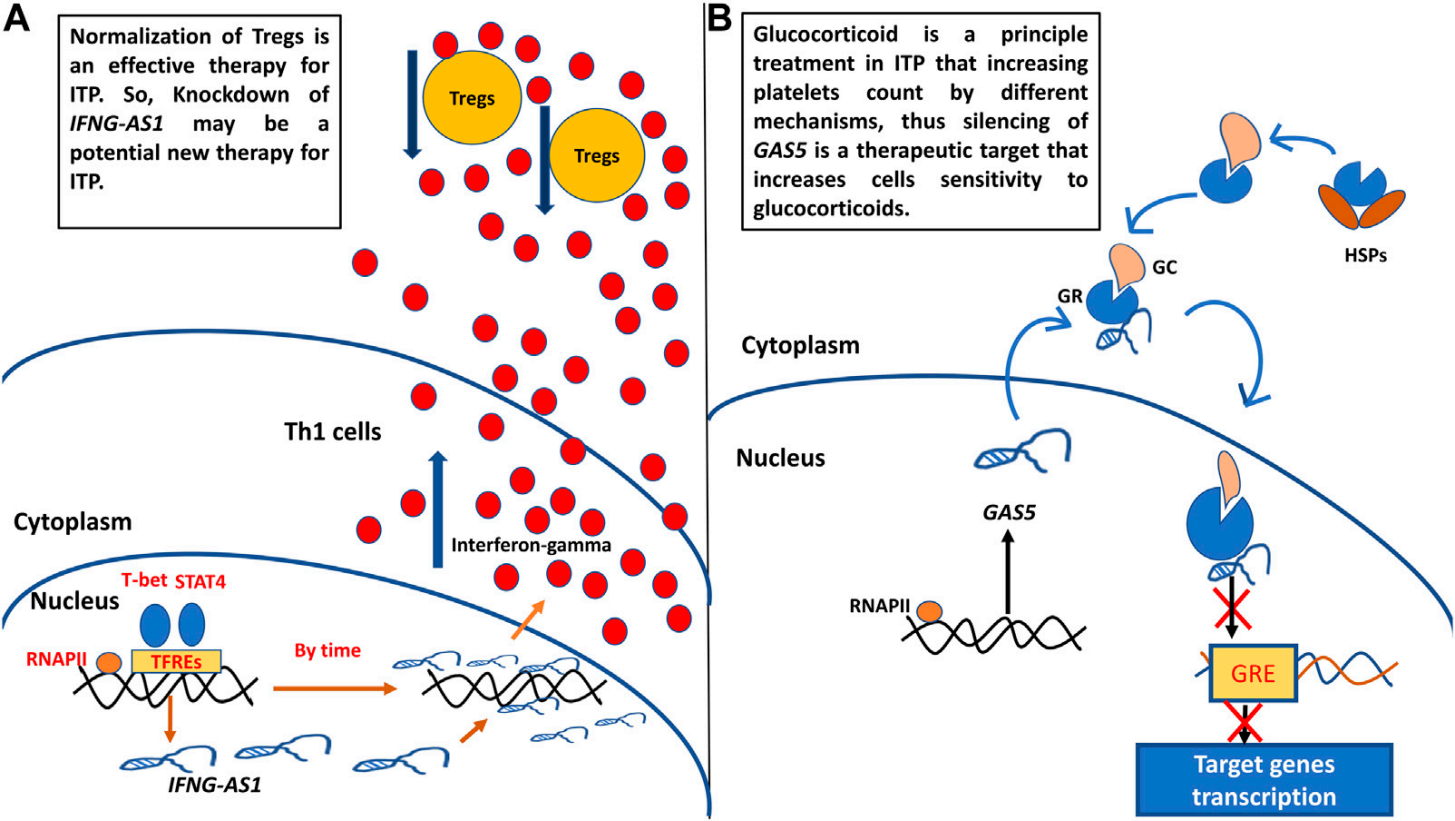
**(A)** Probosed function of *IFNG-AS1* in childhood ITP pathogenesis and its supposed role as target of therapy. **(B)** Proposed function of *GAS5* in childhood ITP pathogenesis and its supposed role as target of therapy. Th1: T helper type 1 cells, Tregs: regulatory T cells, RNAPII: RNA polymerase II, TFREs: transcription factors response elements, GC: glucocorticoid, GR: glucocorticoid receptor, GRE: glucocorticoid response element.

After reviewing the previous literature to explore the functions of target genes in ITP pathogenesis, we designed (Figure 6) to show how these genes could be targets of therapy in childhood ITP.

### Proposed functions of long noncoding RNAs *IFNG-AS1* and *GAS5* in childhood ITP pathogenesis and their supposed roles as targets of therapy

A. We designed Figure 6 to show the proposed function of target genes *IFNG-AS1* and *GAS5* in childhood ITP pathogenesis and their potential usage as targets of therapy. *IFNG-AS1* is selectively expressed in T helper1 (Th1) cells by the effect of Stat4 and T-bet transcription factors (Collier et al., 2012b), upregulated *IFNG-AS1* promoting IFN-γ expression and secretion (Petermann et al., 2019). Excessive IFN-γ decreases CD4^+^CD25+FoxP3+ Treg cell numbers (the major cells that relate to the maintenance of auto-tolerance) (Zufferey et al., 2017). Normalization of Tregs is an effective therapy for ITP (Semple et al., 2010), So, Knockdown of *IFNG-S1* may be a potential new therapy for ITP (Figure 6A).

B. *GAS5* is associated with the glucocorticoid receptors (GRs) through hindering binding of GRs to target genes’ glucocorticoid receptor elements (GREs), and repressed GRs transcriptional activity of endogenous glucocorticoid-responsive genes (Wapinski and Chang, 2011; Kino et al., 2010). Hence, the knockdown in *GAS5* levels may have an impact on the increased sensitivity of immunosuppressive treatment with glucocorticoid-related drugs (Figure 6B).

## Discussion

Childhood Immune Thrombocytopenia (ITP) is typically a benign self-limiting condition that resolves in a few months. However, between 20 and 30% of patients develop chronic ITP, which puts them at risk of serious complications including bleeding such as cerebral hemorrhage and blood cancer (Ayoub et al., 2020). Efficient treatment could decrease the susceptibility of ITP children to these serious complications (Makis et al., 2017) (Kühne et al., 2011). LncRNAs tangled in immune system regulation could be therapeutic targets (Zou and Xu, 2020). Abnormal *IFNG-AS1* and *GAS5* expression levels have been linked to a number of autoimmune disorders, implying that these genes may play a role in the pathogenesis of autoimmunity and could represent new targets of therapy (Wu et al., 2017), (Peng et al., 2020a).

From the facts that; primary ITP provocation is a result of immune system gene dysregulation, lncRNAs are immune gene regulatory molecules, *IFNG-AS1* and *GAS5* are two lncRNAs that are directly linked to autoimmune disorders, previously related to adult ITP and no other previous study tested the expression of these genes in childhood ITP, we construct this case-control study to elucidate expressions of target lncRNAs in childhood ITP and their association with pathophysiology and clinical features of the disease as well as their association with types and treatment.

Our results revealed that the age (mean ± SD) of our patients was 5.1 ± 2·19 years, and there is no sex prediction unlike in adults, the initial platelet count was 9.33 ± 2.51×10^3^/L. these results agree with other studies for age, sex, and initial platelet counts (Talaat et al., 2014), (Diab et al., 2021). In this paper the common clinical presentation was bleeding ∼35% followed by splenomegaly ∼6%, in contrast, a study by ***Ayoub et al, 2020*** showed rates of ∼ 27% for bleeding and ∼ 3% for splenomegaly (Ayoub et al., 2020). We reported that more than 50% of our patients had persistent and chronic ITP while prior studies documented a range between 30% and 40% of patients prone to the persistence of thrombocytopenia after 6 months of diagnosis (Makis et al., 2017), (Ayoub et al., 2020).

It should be stressed that the etiology of ITP is unknown, and on the question of possible precipitating factors, this study found that nearly 6% of our patients have a positive family history, this was consistent with ***Ayoub et al, 2020*** (Ayoub et al., 2020) but less than percentage documented by ***Diab et al, 2021*** which reported 4% of patients had positive family history (Diab et al., 2021). History of *helicobacter pylori* infection accounted for 6% of our cases; other study reported 10% (Ayoub et al., 2020). While nearly 60% experience a period of febrile illness before disease, this was constant with other studies documented that two-thirds of patients had a preceding infection and febrile illness two to 3 weeks before the disease (Ayoub et al., 2020) (Livak and Schmittgen, 2001).

The most important clinically relevant findings were overexpressed lncRNAs *IFNG-AS1* and *GAS5* in the serum of childhood ITP patients [(median (IQR) = 3.08 (0.2–22.39), mean ± SD = 18.37 ± 19.54 for *IFNG-AS1,* and median (IQR) = 4.19 (0.9–16.91) for *GAS5* than controls, Also, significant higher *IFNG-AS1 and GAS5* were linked to patients with positive family history, patients with persistent or chronic ITP than those who are newly diagnosed, patients with no response or partial response to treatment when compared with patients who completely responded to treatment. In addition, *IFNG-AS1 and GAS5* expression were significantly negatively correlated with platelet count after therapy.

Regards *IFNG-AS1*, our results could be explained by the findings of ***Collier et al, 2012*** who implied that *IFNG-AS1* is a part of a larger family of lncRNAs called lincRNAs that positively control gene transcription. They defined *IFNG-AS1* as a Th1-specific lincRNA that promotes the transcription and secretion of the IFN-γ (Collier et al., 2012a). IFN-γ expression abnormalities have been linked to a variety of autoimmune disorders. In ITP patients, plasma IFN-γ levels were shown to be higher, which hastens disease development by lowering CD4^+^CD25+FoxP3+ Treg levels (responsible for preserving self-tolerance by interaction with APC and lowering CD19^+^ B cell and CD8^+^ T cell responses) (Zufferey et al., 2017), (Talaat et al., 2014). Many pieces of research have demonstrated that ITP patients have decreased Tregs, and interestingly, several medications that boost platelet cell number in ITP, such as glucocorticoid, rituximab, and intravenous immunoglobulin (IVIG), appear to do so *via* normalization of Tregs (Zufferey et al., 2017), (Semple et al., 2010), Thus these findings suggesting that silencing of *IFNG-AS1* is a potential target of therapy for ITP. The previously mentioned data explain the significantly higher *IFNG-AS1* levels presented in the partial response or no response patients group and also explain the significant negative correlation between *IFNG-AS1* Levels and platelet number after therapy.

A central contribution to recent work is a study that examined the levels of *IFNG-AS1* in adult thrombocytopenic patients and found the transcript level of *IFNG-AS1* in peripheral blood mononuclear cells (PBMCs) from active adult ITP patients was lower than in healthy controls. Furthermore, researchers discovered a positive correlation between IFN-γ and *IFNG-AS1* transcript levels in PBMCs from healthy controls, as well as a similar change tendency after short-term activation. As a result, researchers speculated that *IFNG-AS1* has been shown to enhance IFN-γ transcription. Furthermore, IFN-γ overexpression had a negative feedback effect on *IFNG-AS1* expression, resulting in lower *IFNG-AS1* levels in adult ITP patients (Li et al., 2016).

Our findings are further supported by studies that investigated *IFNG-AS1* in other autoimmune diseases which revealed that *IFNG-AS1* expression from PBMCs was elevated in Hashimoto’s thyroiditis patients and contributed to Th1 cell response and IFN-γ expression levels which are implicated in the disease’s etiology (Peng et al., 2015). Likewise, lncRNA signatures in ulcerative colitis colonic tissues revealed increased expression of *INFG-AS1* which is function as an enhancer of inflammation through positive regulation of IFN-γ expression. (Padua et al., 2016). In the same way, the transcript level of lncRNA *IFNG-AS1* and its target gene IFN-γ were shown to be higher in the peripheral blood of rheumatoid arthritis patients (RA) than in controls and they were positively linked (Peng et al., 2020b). Additionally, ***Fouad et al, 2022*** revealed that the expression level of *IFNG-AS1* was upregulated significantly in the serum of patients with Behçet disease compared with controls (Fouad et al., 2022b).

Regarding *GAS5,* the widely accepted function of *GAS5* is that it is a tumor-suppressive lncRNA, which is implicated in a wide range of malignancies. Recently, scientists examined levels of *GAS5* in immune-related diseases and elucidated its underlying molecular function, they concluded that the expression level of *GAS5* was aberrant in patients with autoimmune disorders when compared to controls, and in addition to its tumor arresting function, *GAS5* is a potent repressor of the glucocorticoid receptor (GR) (Mayama et al., 2016).

Glucocorticoids (GCs) are a powerful immunosuppressant and are often the essential treatment of inflammatory and autoimmune illnesses, along with preventing rejection in transplanted patients. Several studies indicated that *GAS5 is* associated with glucocorticoid resistance through its direct attachment to the GR protein by competing with glucocorticoid receptor element (GRE) and acting as a decoy GRE, preventing glucocorticoid-induced gene transcription upregulation, and decreasing GCs activity so contributing to the development of numerous autoimmune diseases (Wu et al., 2020) (Suo et al., 2018). Corticosteroids, which are therapeutically variants of the glucocorticoids, are the principal therapy in ITP patients especially in chronic and resistant cases by different mechanisms include; raising the number of circulatory Tregs, reviving the Th1/Th2 proportion, restoring the Th17 count, constant with a rise in IL-10 and TGF- β, also, it alters B cell activation by lowering beta-cell stimulator (BlyS) and modulates dendritic cells (DCs) (Zufferey et al., 2017). Hence, the knockdown in *GAS5* levels may have an impact on increasing the sensitivity of ITP patients to immunosuppressive treatment with glucocorticoid-related drugs. According to these data, we can interpret the significantly higher *GAS5* associated with partial or no response patients group, and the negative correlation between GAS5 expression and platelet number after treatment.

Support for this interpretation comes from ***Moharamoghli et al, 2019*** who found that T cells from RA patients had higher amounts of *GAS5* than those from controls (Moharamoghli et al., 2019), Also, ***Suo et al, 2018*** documented that *GAS5* and *miR21* levels were considerably higher in CD4^+^ T cells from SLE patients than in control subjects, and *GAS5* expression in CD4^+^ T cells was higher in ulcerated SLE patients than in non-ulcerated SLE patients (Suo et al., 2018). In a similar vein, ***Gharesouran et al, 2018*** found that *GAS5* levels were up-regulated in multiple sclerosis patients (Gharesouran, Taheri, Sayad, Ghafouri-Fard, Mazdeh, Davood Omrani).

On the other hand, a recent study documented that *GAS5* expression was downregulated in ITP patients’ PBMCs and ITP mice’s spleen tissues, and overexpression of *GAS5* suppresses Th17 differentiation *in vitro* and relieved ITP *in vivo via* STAT3 reduction. Similarly, ***Li et al, 2019*** concluded that the level of *GAS5* was reduced in RA synovial tissues (Li et al., 2020). Furthermore, *GAS5* and IL-10 mRNA levels in myasthenia gravis patients’ peripheral blood mononuclear cells (PBMCs) were significantly lower than in healthy controls (Peng and Huang, 2022). This inconsistency of results may be due to variances in samples and disease nature.

A strong positive correlation between both genes was detected that confirms their synergistic effects. The ROC curve analysis for lncRNAs *IFNG-AS1* and *GAS5* in ITP patients are, demonstrating the diagnostic utility of these markers as predictors in distinguishing between patients with ITP and controls with sensitivity and specificity reached 100.0% in the case of the combination of both genes.

From the preceding discussion, we speculated that both genes are diagnostic biomarkers for childhood ITP, and the increasing *IFNS-AS1* and *GAS5* expressions in childhood ITP patients may participate in the progress of the disease, especially its higher levels were significantly associated with persistent and chronic ITP patients than newly diagnosed patients. This finding agreed with ***Li et al, 2016*** who documented associated high *IFNG-AS1* with active ITP when compared to inactive ITP patients (Li et al., 2016) Also, high *IFNG-AS1* was present in active ulcerative colitis (Padua et al., 2016) and high *GAS5* was linked to active SLE (Suo et al., 2018). Thus, *IFNG-AS1* and *GAS5* may be used as predictors of the course of the disease.

This study confirms that *IFNG-AS1* and *GAS5* may be potential targets of therapy as we found that higher target genes were detected in patients with no or partial response to treatment when compared to patients who completely responded to treatment, these results further support the idea of *GAS5* function as glucocorticoid resistant enhancer (Mayama et al., 2016). In addition, we conveyed that *IFNG-AS1* and *GAS5* expressions were significantly negatively correlated with platelet count after therapy, this result was consistent with ***Li et al, 2016*** who documented a negative correlation of *IFNG-AS1* with platelet count, but they found no correlation with treatment or duration of the disease (Li et al., 2016).

The limitations of the current study include; 1) The sample size of the current study was modest and selected from the same geographical area which provoked the possibility of selection bias. 2) Insufficiency of scientific literature demonstrating the precise function of target lncRNAs in general and in particular to pediatric diseases including childhood ITP. Further multicentric studies are needed to confirm our findings, verify the utility of target genes as targets of therapy, and better understand the molecular role of *IFNG-AS1* and *GAS5* in the etiology and pathogenesis of childhood ITP.

The current study contains several new and important insights; findings suggest that lncRNAs *IFNG-AS1* and *GAS5* are novel diagnostic and prognostic biomarkers for childhood ITP that can aid in a precise prediction of the disease’s progress at the time of diagnosis and could be a useful tool for treatment planning, reducing the risk of bleeding while avoiding drug side effects. We speculated that both genes have underlying molecular contributing roles in the development and prognosis of childhood ITP and may be used as targets of therapy to reduce the propagation of the disease to a chronic state.

## Conclusion

LncRNAs *IFNG-AS1* and *GAS5* are novel diagnostic and prognostic genetic markers for childhood ITP.

## Data Availability

The original contributions presented in the study are included in the article/supplementary material, further inquiries can be directed to the corresponding author.
